# Older LGBTQIA+ adults: Analyzing shame and double stigmatization

**DOI:** 10.1192/j.eurpsy.2025.1764

**Published:** 2025-08-26

**Authors:** S. von Humboldt

**Affiliations:** William James Center for Research, ISPA – Instituto Universitário, Lisbon, Portugal

## Abstract

**Introduction:**

Discriminatory and abusive behaviors may strongly affect older LGBTQIA+ (lesbian, gay, bisexual, transgender, queer or questioning, intersex, asexual, and more) adults, and poorer health is linked to discriminating practices. Shame is associated with ageism and sexual orientation-based discrimination and may be a significant impediment to aging well by undermining older adults’ sense of agency, health, and well-being. However, little is still known about older LGBTQIA+ adults who encounter double stigmatization.

**Objectives:**

This study aims to explore the dimensions of shame and double stigmatization of older LGBTQIA+ adults.

**Methods:**

The significant psychological challenges and relevant themes experienced by older LGBTQIA+ adults, as related to shame and double stigmatization, were illustrated by semi-structured interviews with 329 older adults in a qualitative study through content analysis.

**Results:**

The results highlight four themes of shame and double stigmatization among older LGBTQIA+ adults, encompassing (1) ageism (86.5%), (2) sexual orientation-based discrimination (81.1%), (3) stereotyping (76.8%), and (4) social exclusion (72.3%).

**Conclusions:**

The study reveals the profound negative effects of shame and double stigmatization on older LGBTQIA+ adults, emphasizing the need for inclusive policies and interventions to address multiple forms of discrimination and promote equitable treatment in aging populations.

Keywords: Shame; Ageism; double stigmatization; LGBTQIA+; multiple discrimination; older adults.

**Disclosure of Interest:**

None Declared

